# Chronic Hepatitis B Virus Persistence: Mechanisms and Insights

**DOI:** 10.7759/cureus.78944

**Published:** 2025-02-13

**Authors:** Samrita Naidu, Severine Margeridon

**Affiliations:** 1 Virology, Rio Americano High School, Sacramento, USA; 2 Molecular Diagnostics and Assay Development, Bio-Rad Laboratories, San Francisco, USA

**Keywords:** chronic hepatitis b infection, hepatitis b functional cure, hepatitis b infection, hepatitis b virus, viral persistence

## Abstract

Chronic hepatitis B (CHB) virus infection can lead to severe liver diseases, including cirrhosis and hepatocellular carcinoma. The chronicity of the hepatitis B virus (HBV) occurs because of the persistence of viral covalently closed circular DNA (cccDNA) within hepatocytes. The cccDNA serves as the template for viral replication and is central to HBV, maintaining a viral reservoir within the host. Despite therapeutic advancements, eliminating cccDNA remains elusive due to its evasion of immune surveillance. This review explores the formation and maintenance of cccDNA, highlighting host factors influencing cccDNA stability and viral replication. It also discusses current treatment strategies, including interferon-based therapies and nucleoside/nucleotide analogs, which aim to suppress viral replication. Emerging therapies such as gene editing and molecular interventions hold promise for targeting cccDNA directly. Currently, research is focused on making medications that target host factors of interest to disrupt or clear the viral reservoir. However, future research should focus on innovative approaches that directly target the cccDNA minichromosome, aiming for sustained viral suppression and potentially a cure for the HBV infection.

## Introduction and background

**Figure 1:**
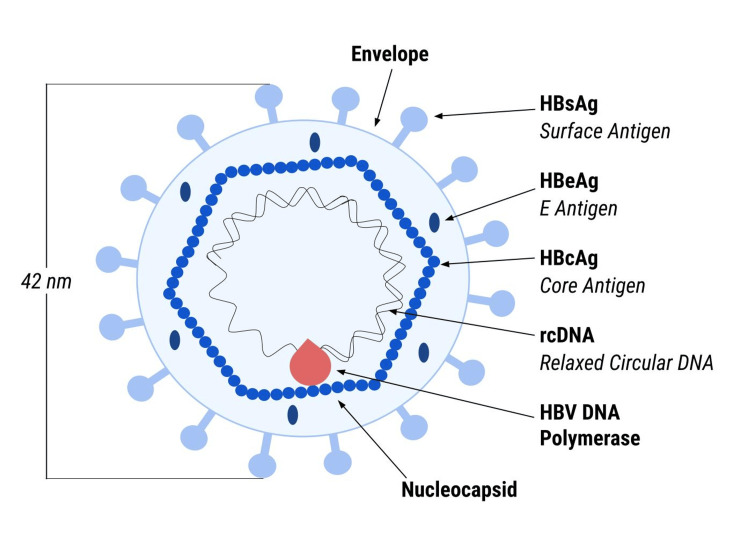
Hepatitis B virion. HBV: hepatitis B virus; HBeAg: hepatitis B virus E antigen; HBsAg: hepatitis B surface antigen; HBcAg: hepatitis B virus core antigen; rcDNA: relaxed circular DNA.

Hepatitis B virus (HBV) poses a substantial global health issue affecting approximately 3.2% of the world's population in 2022 [1]. The prevalence and persistence of HBV remain a significant global health challenge as the World Health Organization (WHO) estimated that 254 million people were living with chronic hepatitis B (CHB) in 2022 and are at a higher risk for developing other liver diseases, including cirrhosis and hepatocellular carcinoma [2]. Hepatitis B is a partially double-stranded circular DNA virus that belongs to the *Hepadnaviridae* family [3]. The viral particle consists of double-stranded DNA and a polymerase (Figure 1). The icosahedral protein nucleocapsid holding these inside is made of proteins known as hepatitis B virus core antigen (HBcAg), which is wrapped by an envelope. Hepatitis B virus E antigen (HBeAg), a common indicator for active viral replication, is found between the nucleocapsid and envelope. Persistent CHB infection occurs due to the maintenance of a viral circular episomal DNA called covalently closed circular DNA (cccDNA) within the liver. cccDNA is crucial to the life cycle and progeny generation of HBV because cccDNA is the template for the transcription of all viral RNAs, including pregenomic RNA (pgRNA). PgRNA uses viral reverse transcription to form more copies of cccDNA eventually [4]. Though treatments can reduce viremia by inhibiting viral replication, the persistence of cccDNA within hepatocytes, impaired immune response, and other causes pose obstacles to finding a cure for HBV [5]. This comprehensive review will identify how cccDNA is formed and how it persists within hepatocytes, the function of cccDNA in CHB infections, the host factors that regulate the cccDNA reservoir within an organism, and treatment approaches.

## Review

Formation of cccDNA

HBV Genome

The relaxed circular DNA (rcDNA) genome of HBV is partially double-stranded and is approximately 3,200 base pairs in length. It contains four overlapping reading frames (ORFs): S, C, P, and X. The S ORF produces the viral surface envelope protein known as hepatitis B surface antigen (HBsAg), which is divided into pre-S1, pre-S2, and S regions. The C ORF encodes for the hepatitis B virus core antigen (HBcAg) and hepatitis B virus E antigen (HBeAg), depending on the start position of translation. The P ORF encodes the polymerase that is functionally divided into three domains: the terminal protein domain, which is involved in encapsidation and initiation of minus-strand synthesis; the reverse transcriptase (RT) domain; and the ribonuclease H domain (RNaseH), which also allows for the synthesis of the second strand of cccDNA. The HBV X ORF produces hepatitis B virus X antigen (HBxAg), which is involved in signal transduction, transcriptional activation, DNA repair, and inhibition of protein degradation [6].

HBV Replication Cycle

**Figure 2:**
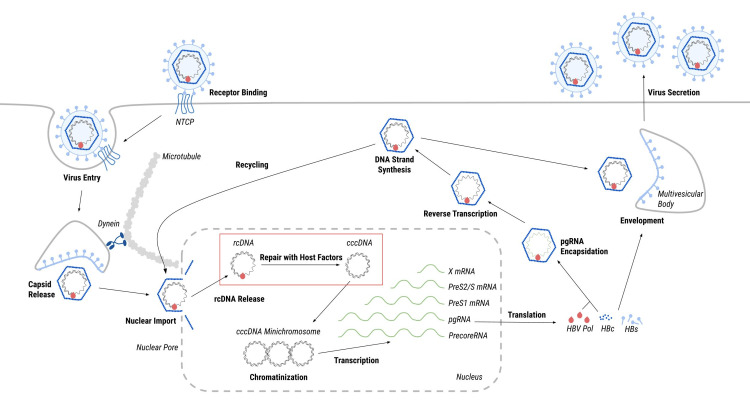
Replication cycle of cccDNA. The green strands represent RNA and the black strands represent DNA. The red box highlights the step in which cccDNA is formed from rcDNA. cccDNA: covalently closed circular DNA; rcDNA: relaxed circular DNA; NTCP: sodium taurocholate cotransporting polypeptide; pgRNA: pregenomic RNA; HBV: hepatitis B virus.

The unique replication cycle of HBV is important to understand how cccDNA is formed. The replication cycle of HBV begins as the viral capsid binds to the bile acid transporter, sodium taurocholate cotransporting polypeptide (NTCP), on the surface of a hepatocyte, and the HBV virion is internalized through receptor-mediated endocytosis (Figure 2). Using the intracellular microtubule system, the viral capsid travels through the cytoplasm to the nuclear pore. A small, partially double-stranded, rcDNA is released in the nucleus. RcDNA is then converted into cccDNA, which becomes chromatinized. CccDNA is the template for the transcription of all HBV RNAs, each of which encodes for specific antigens used to build HBV. In the cytoplasm, pgRNA is enclosed in capsids and reverse-transcribed into rcDNA. The resulting capsids are either enveloped at the multivesicular body (MVB) within the cell and released as new virions, or transported back to the nuclear pore to increase the reservoir of intracellular cccDNA [4,6].

CccDNA Formation

For the viral genome to be expressed, rcDNA must convert into cccDNA before RNA transcription. RcDNA cannot be transcribed because of four distinct lesions: in the first lesion, HBV polymerase (POL) is covalently attached to the 5′ end of the minus strand with a tyrosyl phosphodiester bond; second, the terminal redundancy sequence includes a 10-nucleotide DNA flap on the minus strand; third, a 5′-capped RNA primer; and fourth, a single-stranded DNA (ssDNA) gap on the plus strand [6]. Converting rcDNA to cccDNA, which involves host DNA repair machinery, is a critical step in the HBV life cycle (Figure 2) [7-9].

CccDNA formation starts with the liberation of HBV POL from rcDNA, facilitated by multiple mechanisms. The minus strand is repaired by cleavage of terminally redundant sequences by nucleases. DNA ligase 1 (LIG1) or DNA ligase 3 (LIG3) bind to the nicked region and attach the cleaved sequences. The positive strand is repaired with the aid of DNA polymerase κ, along with polymerases α, δ, and ɛ, and DNA topoisomerases I and II. DNA ligase 1 (LIG1) and DNA ligase 3 (LIG3) facilitate the ligation of both strands [6,9]. Finally, chromatinization of the DNA occurs, which involves histone proteins and results in the formation of cccDNA [10]. This chromatinization creates a viral minichromosome, which emulates the structure of our chromosomes with histones and other host proteins. The creation of the minichromosome is the key to cccDNA long-term persistence within hepatocytes and HBV chronicity [11,12]. Many host factors are implicated in the promotion and repression of this cccDNA.

Some drugs in development, gene therapies, and small molecule inhibitors treat HBV by targeting host factors promoting the maintenance of this viral minichromosome [13]. If these medications become available to consumers, they could lessen the viral load of individuals with HBV immensely. However, the most direct and efficient approach to a cure for HBV would be targeting the viral minichromosome, and research should be more directed toward this [14].

Host determinants influencing cccDNA stability

Host Factor Interactions With CccDNA

Many host factors regulate the expression of HBV cccDNA. Promoter and enhancer elements of cccDNA contain sites where both liver-specific and widely distributed transcription factors and nuclear receptors can bind. This is the main method by which host factors positively regulate cccDNA activity. Several transcription factors and epigenetic modifiers suppress the cccDNA activity leading to a reduction in viral replication, a host defense mechanism [15].

Wong et al. defined the functional cure of HBV as “sustained undetectable circulating HBsAg and HBV DNA after a finite course of treatment” [16]. This goal of the scientific community has not yet been achieved because of the persistence of cccDNA. The following compilations of noteworthy host factors are the first step to creating more treatments for HBV and to achieving this goal. Tables 1, 2 show the various host factors that impact the activation and inhibition of cccDNA.

**Table 1:** Host factors that promote cccDNA expression. cccDNA: covalently closed circular DNA.

Function	Host factors
Transcription factors	HNF1, HNF3/FoxA, C/EBP, NF1, SP1, AP-1, TBP, CREP, Oct1, NFR1 [15], TFIIB, CREB, KLF15, LRH-1, YBX1 [17]
Nuclear receptors	HNF4, RXR𝜶/PPAR𝜶, FXR [15]
Transcriptional coactivator	CRTC1 [15]
Acetyltransferases (HATs)	CBP, P300, PCAF/GCN5 [15], KAT2A [18]
Histone demethylase	LSD1 [15]
Lysine methyltransferase (KMT)	Set1A [15]
Protein and pre-mRNA splicing factor	PRPF31 [19]
Nuclear transcription factor	NF-Y [17]
Cytokinesis protein	DOCK11 [20]
Long non-coding RNA	DLEU2 [21]
Phosphodiesterase	TDP2 [15]
Chromatin-binding protein	HMGN1 [22]
Deacetylase	SIRT2 [23]
Protein kinase	PRKDC [24]

**Table 2:** Host factors that repress cccDNA expression. cccDNA: covalently closed circular DNA; lncRNA: long non-coding RNA.

Function	Host factors
Transcription factors	NF-κB, PROX1, YY1, ZHX2, STAT1/3, ZEB2, P53, RFX1, HOXA10, ATF2 [17], CTCF [25], MafF [26], HOXA-AS2 [27]
Lysine methyltransferases (KMT)	EZH2, SETDB1 [15]
Class III histone deacetylases (HDAC)	SIRT1, SIRT3 [15]
Protein arginine methyltransferases (PRMT)	PRMT1, PRMT5 [15]
Class I histone deacetylase (HDAC)	HDAC1 [15]
Structural maintenance of chromosomes	Smc5/6 [15]
DEAD-box RNA helicase	DDX3 [15]
Cytidine deaminases	APOBEC3A, APOBEC3B [15]
Nuclear receptor	SHP [17]
Nuclear factor	HNF6 [17]
Interferon-induced GTP-binding protein	MX2 [28]
Cohesin complex	SMC3 [29]
Protein kinase	CaMKII [30]
Interferon	IFN-α [31]
Receptor	TR4 [32]
Intrahepatic homeobox protein	MSX-1 [33]
Nuclear lncRNA	LINC01431 [34]

Another group of factors, the CCAAT-enhancer-binding protein family, has been found to positively and negatively regulate cccDNA expression, based on its own expression levels. The TRIM family of proteins and Mx proteins also suppress HBV DNA [17,35].

CccDNA Dilution During Mitosis

**Figure 3:**
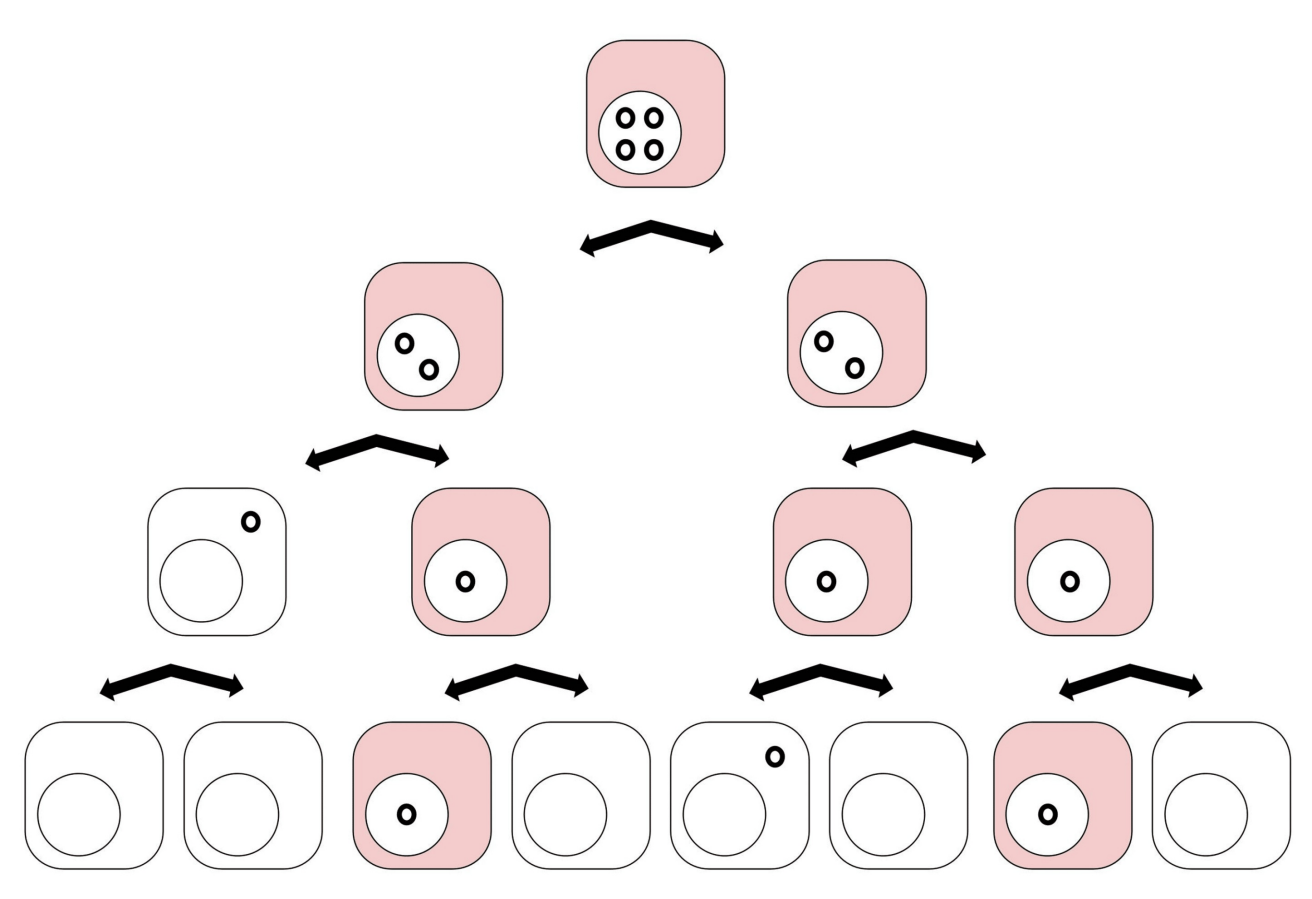
CccDNA dilution during mitosis. The smaller black circles represent cccDNA within the cells. The pink cells are infected while the white cells are uninfected. This demonstrates that as cells divide, the cccDNA reservoir decreases. cccDNA: covalently closed circular DNA.

The HBV cccDNA load is decreased when a cell undergoes mitosis. When an infected primary human hepatocyte (PHH) divides, the cccDNA load is distributed among the daughter cells. Eventually, the re-forming nucleus excludes cccDNA. Therefore, PHH mitosis consequentially leads to cccDNA dilution and cccDNA loss (Figure 3) [36,37]. In a study done in 2018, cryopreserved PHHs from four different donors were infected with HBV. Xia et al. found that HBV infection changes the expression of various growth factors and deregulates cell cycle pathways [38]. Additionally, in a sophisticated in vivo study in which HBV-infected PHHs were allowed to proliferate in the liver of human chimeric mice, it was found that HBV cccDNA underwent a profound decrease after cell division. However, sporadic quiescent PHHs prevented complete viral clearance [39]. Another study explored how the HBx protein (hepatitis B virus regulatory protein X) affects liver regeneration in two separate strains of HBx transgenic mice. Their findings suggested that the HBx protein exerted significant growth arrest in hepatocytes, disrupting normal cell-cycle progression, causing failure of liver functionality, and leading to abnormal cell death [40]. Many other studies have supported the claim that HBV deregulates cell cycle pathways and alters the expression of growth factors, which is one of the many strategies that HBV employs to ensure that cccDNA remains within hepatocytes.

Host immune response in targeting HBV

According to a 2022 study, the global prevalence of HBV was 3.2%. Adult acute HBV typically results in chronic hepatitis B (CHB) in fewer than 5% of cases, whereas infection acquired during infancy and early childhood results in CHB in approximately 95% of cases [1]. These statistics show that the host immune response, particularly involving natural killer (NK) cells and T-cells, plays a crucial role in clearing HBV infection from the body. The adults’ stronger immune systems are largely able to keep the HBV infection from reaching a state of chronicity. On the other hand, infants, with weaker immune systems, more commonly progress to CHB. Studies have also demonstrated that in acute HBV infection, the T-cell responses to HBV proteins are strong, polyclonal, and multi-specific, whereas in CHB, immune responses are weaker [41].

Natural Killer Cells

Host immune responses can decrease cccDNA levels in the liver through the removal of infected hepatocytes by NK cells and cytotoxic T lymphocytes [15]. In an acute HBV infection, the hasty production of interferon-1 (IFN-1) characterizes the innate immune response. This triggers many antiviral mechanisms, notably the upregulation of major histocompatibility complex 1 (MHC 1) of infected cells. The innate immune response also consists of the production of interferon-beta (IFN-β) and other interferon-stimulated genes (ISGs), which help clear cccDNA without causing cell death [42]. In a study, a significant reduction in cccDNA was found in acutely HBV-infected chimpanzees long before the peak of T-cell infiltration, supporting the claim that noncytopathic antiviral mechanisms contribute to viral clearance during acute hepatitis B [43]. In a study investigating the incubation phase of acute hepatitis B, it was found that the maximum number of circulating NK cells was observed early in the incubation period of patients with acute HBV infection [44]. A different study demonstrated that NK cell activation is significantly suppressed in individuals with acute HBV infection compared to healthy subjects, particularly during peak viremia [45]. In conclusion, these findings suggest that NK cells play a crucial role in acute HBV infection, although their functionality is suppressed. HBV suppresses NK cells through various mechanisms, including phenotype modification. HBV infection can change the phenotype of NK cells by downregulating activating receptors (NKG2D, 2B4, NKp30) and upregulating the inhibitory receptor (NKG2A) [46]. More methods of HBV suppression of NK cells are discussed in the next paragraph in the context of CHB.

In CHB, several studies indicate that NK cells show specific defects in their ability to combat viruses. This “functional dichotomy,” a term coined for this phenomenon by Oliviero et al. [47], consists of reduced or increased cytolytic activity and lowered cytokine production. In an experiment where monocytes and NK cells were co-cultured, it was observed that HBV-induced suppressive monocytes caused NK cells to differentiate into regulatory NK cells (NK-regs). These NK-regs expressed anti-inflammatory cytokines such as interleukin-10 (IL-10) [48]. High levels of IL-10 create an immunosuppressive cytokine environment that may hinder NK cells from producing interferon-gamma (IFN-γ). IFN-γ inhibits various steps in the virus replication cycle [49]. In a study involving 21 acutely infected patients, researchers concluded that the suppression of NK cells corresponded with increased levels of both IL-10 and HBV viremia [45].

Another defect in NK cells is that they may negatively regulate HBV-specific CD8+ T-cells. This occurs because HBV-induced NK-regs suppress HBV-specific T-cell function by decreasing T-cell proliferation and inhibiting NK cells' antiviral function directly [50]. This claim is supported by multiple studies in which the researchers reported that removing NK cells enhances the antiviral activity of CD8+ T-cells [51,52].

CD4+ and CD8+ T-cells

The immune responses of HBV-specific CD4+ and CD8+ T-cells are essential for managing HBV pathogenesis. In acute HBV infection, HBV-specific CD8+ T-cell responses correlate with viral clearance. Using a transgenic mouse model, a study showed that viral replication can be halted by inflammatory cytokines, IFN-𝛾 and tumor necrosis factor (TNF), secreted from HBV-specific CD8+ T-cells while killing only a small fraction of hepatocytes [53]. The contribution of non-cytolytic effector mechanisms has been additionally validated via a cell culture model and in acutely infected chimpanzees [42].

In CHB, T-cell exhaustion manifests as diminished or absent virus-specific T-cell responsiveness. This includes reduced effector cytotoxicity, decreased cytokine secretion capacity, and persistent expression of inhibitory receptors such as programmed cell death-1 (PD-1), lymphocyte activation gene-3 (TIM-3), cytotoxic T lymphocyte-associated antigen-4 (CTLA-4), and cluster of differentiation 244 (CD244) protein. T-cell immune tolerance and specific T-cell exhaustion occur because of long-term exposure to a high concentration of viral antigens [50,54].

Other notable cells that aid the host immune response include monocytes, macrophages, and neutrophils. HBsAg, the HBV surface antigen, induces monocyte immunosuppression through a specific signaling pathway. Kupffer cells (KCs), a type of macrophage, reside in the liver and promote antiviral immunity by increasing their production of certain cytokines. Neutrophils combat viruses mainly by employing reactive oxygen species (ROS), phagocytosis, degranulation, and neutrophil extracellular traps (NETs). Neutrophils produce interleukin-12 (IL-12), which in turn promotes NK cell secretion of more interferons. This enhances cytotoxic T-cell activity. HBcAg and HBeAg inhibit neutrophil activation and migration to the infection site, leading to T-cell dysfunction and persistent HBV infection [55].

FDA-approved treatments

Currently, several medications have been approved for the treatment of CHB: two subcutaneous interferon (IFN)-based drugs and seven oral nucleos(t)ide analogs (NUC), as given below in Table 3.

**Table 3:** FDA-approved treatments for chronic hepatitis B.

Treatment	Administration	Drug type
Interferon alpha (IFN-𝛼) [56]	Subcutaneous	Interferon-based
Pegylated interferon (Peg-IFN) [57]	Subcutaneous	Interferon-based
Tenofovir alafenamide (TAF) [58,59]	Oral	Nucleoside reverse transcriptase inhibitor
Tenofovir disoproxil fumarate (TDF) [59]	Oral	Nucleotide analogue reverse transcriptase inhibitor
Entecavir (ETV) [60]	Oral	Nucleoside reverse transcriptase inhibitor
Telbivudine (LdT) [61]	Oral	Thymidine nucleoside analogue
Adefovir (ADV) [62]	Oral	Nucleotide analogue reverse transcriptase inhibitor
Lamivudine (LAM) [63]	Oral	Nucleoside reverse transcriptase inhibitor
Emtricitabine [64]	Oral	Nucleoside reverse transcriptase inhibitor

Interferons

Interferons (IFNs) are cytokines, soluble glycoproteins with potent antiviral activities. Interferon-α (IFN-α) is produced by plasmacytoid dendritic cells and is typically administered subcutaneously three times weekly to treat HBV. The pegylation of IFN-α extends its half-life, enabling a once-weekly dose of treatment.

IFN affects different stages of the HBV life cycle, including virus entry, uncoating, transcription, translation, and assembly. It hinders HBV replication by reducing RNA transcription. IFN-α also boosts the production of APOBEC3, a cytidine deaminase, prompting hypermutations in HBV DNA, which in turn hinder viral replication. Additionally, IFN-α can activate immune responses through cells that target infected liver cells, reducing the number of cells containing cccDNA [55,56].

Nucleoside/Nucleotide Analogs

Nucleos(t)ide analogs work by converting into active triphosphate metabolites. Then these molecules are incorporated into the developing viral DNA chain during replication, causing chain termination and inhibition of viral replication [65].

Treatment approaches in development

The treatment approaches in development are presented in Table 4 [13].

**Table 4:** Treatment approaches in development. Source: Ogunnaike et al. [13]. HBV: hepatitis B virus; pgRNA: pregenomic RNA; cccDNA: covalently closed circular DNA; HBsAg: hepatitis B surface antigen.

Treatment	Mode of action
Entry inhibitors	Compete with HBV virions for the binding site of the sodium taurocholate cotransporting polypeptide (NTCP) receptors on hepatocytes. Ex: Bulevirtide (Myrcludex-B).
Capsid assembly modulators	Inhibit HBV replication by targeting pgRNA encapsidation and blocking early viral life cycle stages, including cccDNA formation.
Immunomodulators - toll-like receptor agonists, monoclonal antibodies, checkpoint inhibitors, and therapeutic vaccines	Enhance the HBV-specific immune response.
Gene therapies - RNA interference (RNAi)	Inhibit HBV gene expression by destabilizing or inhibiting HBV mRNA, modulating host proteins, and targeting cccDNA causing mutation. RNAi targets post-transcriptional activities of HBV RNA transcripts.
Small molecule inhibitors	Reduce levels of viral proteins and viremia by interfering with viral RNA, HBsAg production, and host factors involved in HBV replication.

Gene Editing and Molecular Interventions

Initially identified as a bacterial immune defense system, clustered regularly interspaced short palindromic repeats (CRISPR) technology has emerged as a promising tool for permanently suppressing HBV expression with specificity. The CRISPR/Cas9 (CRISPR-associated protein 9) system has shown efficient capability in inducing mutations within HBV DNA resulting in the suppression of HBV replication. This system could offer a novel therapeutic approach for HBV infection [66].

## Conclusions

CccDNA persistence within hepatocytes has been identified to stem from its stable minichromosome structure and the variety of host factors it interacts with. However, a combination of host factors, immune response, and cccDNA dilution can keep this infection from reaching a stage of chronicity in most adults with acute HBV. This review identified host factors of interest as potential targets for treatment. This review also highlighted that making many treatments targeting an array of host factors and certain parts of the viral replication cycle is vital. However, research efforts should focus more on developing a treatment that targets the cccDNA minichromosome directly to stop its ability to transcribe viral RNAs. This approach may be one of the most promising pathways toward achieving a functional cure for HBV.
